# Body Mass Index Is Associated with Hypercholesterolemia following Thyroid Hormone Withdrawal in Thyroidectomized Patients

**DOI:** 10.1155/2014/649016

**Published:** 2014-07-09

**Authors:** Dong Yeob Shin, Kwang Joon Kim, Yongin Cho, Kyeong Hye Park, Sena Hwang, Woong Youn Chung, Eun Jig Lee

**Affiliations:** ^1^Division of Endocrinology and Metabolism, Department of Internal Medicine, Yonsei University College of Medicine, 50 Yonsei-ro, Seodaemun-gu, Seoul 120-752, Republic of Korea; ^2^Department of Medicine, The Graduate School, Yonsei University College of Medicine, 50 Yonsei-ro, Seodaemun-gu, Seoul 120-752, Republic of Korea; ^3^Severance Executive Healthcare Clinic, Yonsei University Health System, 50 Yonsei-ro, Seodaemun-gu, Seoul 120-752, Republic of Korea; ^4^Severance Check-up, Yonsei University Health System, 10 Tongil-ro, Jung-gu, Seoul 100-753, Republic of Korea; ^5^Department of Endocrinology and Metabolism, National Health Insurance Service, Ilsan Hospital, Goyang, Gyeonggi 410-719, Republic of Korea; ^6^Department of Surgery, Yonsei University College of Medicine, 50 Yonsei-ro, Seodaemun-gu, Seoul 120-752, Republic of Korea

## Abstract

Thyroid hormone withdrawal (THW) for postoperative radioiodine adjuvant therapy or diagnostic radioiodine whole body scan in patients with differentiated thyroid cancers results in acute thyroid hormone deficiency and abnormal lipid profiles. To better clarify the clinical pattern of dyslipidemia occurring after THW, we retrospectively analyzed the association between serum total cholesterol level after THW and various clinical factors in a total of 61 patients who underwent total thyroidectomy due to papillary thyroid cancers from January 2010 to March 2012, in Severance Hospital, Seoul, Korea. Preoperative baseline total cholesterol was significantly correlated with post-THW total cholesterol level; however, age, gender, or elevated TSH level after THW itself was not correlated with post-THW total cholesterol level. A significant correlation between preoperative measured BMI and post-THW total cholesterol level was found (*r* = 0.263, *P* = 0.041). In multiple logistic analysis, BMI was an independent determining factor of post-THW total cholesterol level (*P* = 0.012).

## 1. Introduction 

Hypothyroidism causes adverse effects on lipoprotein metabolism. The association between thyroid function and dyslipidemia is well demonstrated not only in overt hypothyroidism, but also in low normal thyroid function with TSH level within normal range [1]. Patients with hypothyroidism are known to have significantly higher serum total cholesterol, low-density lipoprotein cholesterol (LDL-C), and triglyceride levels compared to healthy population. In addition, carotid intima-media thickness and flow-mediated vasodilatation, which are surrogated markers of atherosclerosis and vascular endothelial dysfunction, are also known to be associated with thyroid function, even when TSH level is within normal reference range [2–5].

Although dyslipidemia and atherosclerosis are both well-defined major risk factors of cardiovascular diseases, whether patients with high or high normal TSH level are definitely at an increased risk of cardiovascular diseases is still under controversy [6, 7]. The possible influence of various clinical factors related to cardiovascular diseases, such as age, gender, and body mass index (BMI), is one of the reasons contributing to the uncertainty of the association [6, 8]. This suggests that a variety of influencing factors must be taken into account when approaching dyslipidemia—and furthermore, cardiovascular risk factors—accompanied by hypothyroidism.

When performing postoperative radioiodine adjuvant therapy or diagnostic radioiodine whole body scan in patients with differentiated thyroid cancers (DTC), a TSH elevation higher than 30 mU/L achieved through thyroid hormone withdrawal (THW) (withdrawal of levothyroxine (L-T4)) is required in most cases [9]. THW inevitably results in acute thyroid hormone deficiency in patients who have undergone total thyroidectomy, and abnormal lipid profiles, such as hypercholesterolemia, are accompanied in majority of such cases. Several previous studies have investigated hypothyroid symptoms and metabolic effects following THW in patients with DTC; however, none has elucidated the factors influencing post-THW dyslipidemia. A subgroup of such patients actually does not develop dyslipidemia after THW [10–13].

This study retrospectively analyzed the association between serum cholesterol level and various clinical factors in the setting of TSH elevation in order to better clarify the clinical pattern of dyslipidemia occurring after THW in DTC patients and, as a result, found that BMI is an independent factor determining post-THW serum cholesterol level.

## 2. Materials and Methods 

### 2.1. Study Subjects and Clinical Data

A total of 142 consecutive patients diagnosed with papillary thyroid carcinoma with intermediate recurrence risk based on 2009 American Thyroid Association (ATA) guideline and who underwent postoperative 1.1 GBq radioiodine (131-I) remnant ablation (RRA) in Endocrinology Cinic, Severance Hospital, Seoul, Korea, from January 2010 to March 2012, were retrospectively reviewed. Among them, the data of 61 patients who consented to the measurement of lipid profiles following THW were selectively included in the analysis. The lipid profiling was performed simultaneously with stimulated thyroglobulin (Tg) assay when the patients visited the endocrinology clinic after a 3-week THW regime. All subjects who had histories of diabetes mellitus, use of lipid-lowering drugs, or underlying thyroid dysfunction were excluded in the analysis.

The medical records of the patients were reviewed, and the preoperative baseline characteristics, including age, gender, BMI, various biochemical parameters measured before and after surgery, and postoperative RRA, were retrospectively analyzed. This retrospective study was approved by our Institutional Review Board, and informed consent was not required.

As per postoperative follow-up protocol for DTC in our institution, the patients received low-dose RRA 3 months after undergoing total thyroidectomy, and TSH-suppressive dose of L-T4 (150 *μ*g/d) was administered for 3 months after the surgery. After 4 weeks of THW, thyroid function test (TFT) and lipid profiling were performed in conjunction with measurement of Tg, anti-Tg antibody immediately before the oral administration of 1.1 GBq I-131. A postablation whole body scan was performed 48 h after administration of I-131, and, thereafter, TSH-suppressive dose of L-T4 was begun again in all patients.

Following postoperative RRA, the patients visited our endocrinology outpatient clinic approximately every six months during immediate postoperative follow-up periods for surveillance of recurrence and persistence of the disease. The mean duration from the day of RRA to the first and second follow-up visit days was 5.9 ± 1.0 and 15.9 ± 3.7 months, respectively, and a TSH-suppressive does of L-T4 (150 *μ*g/d) was maintained in all patients during this period. In the first follow-up visit after RRA (P2), TFT and lipid profiling were performed in all 61 subjects, and, in 41 subjects, the measurements were repeated in the second follow-up visit after RRA (P3).

### 2.2. Thyroid Function Test and Biochemical Measurements

Baseline TFT and biochemical parameters including plasma glucose, uric acid, total cholesterol, triglycerides, and high-density lipoprotein cholesterol (HDL-C) were measured immediately before total thyroidectomy. TFT and lipid profiles were measured after THW for postoperative RRA and at one or two follow-up visits thereafter.

After 12 h of fasting, all blood samples were obtained from the patients and stored at −70°C for subsequent assays. Serum TSH and free T4 and T3 were measured by chemiluminescent microparticle immunoassay (Architect System, Abbott Ireland Diagnostic Division, Lisnamuck, Longford, Co. Longford, Ireland). Plasma glucose, uric acid, triglycerides, total cholesterol, and HDL-C levels were assayed using a routine Hitachi 7600 autoanalyzer (Hitachi Instruments Service, Tokyo, Japan). Low-density lipoprotein cholesterol (LDL-C) levels were calculated using the Friedewald formula [14].

### 2.3. Statistical Analysis

Data were shown as means ± standard deviations if they were continuous variables. The normal distribution of each variable was examined using Kolmogorov-Smirnov test. The differences between the mean level of baseline total cholesterol of subjects and that of each serial time point were validated through paired sample* t-*test. Spearman correlations were used to examine the relationships between total cholesterol level after THW and age, gender, BMI, TSH level, LDL-C level after THW, and total cholesterol at other time points. Comparison of categorical variables between BMI groups was performed using chi-square test and Fisher's exact test (two-tailed), as is appropriate. To investigate the association between BMI and other clinical parameters including age, gender, and post-THW total cholesterol more accurately, we employed a logistic regression analysis. *P* values < 0.05 were considered statistically significant for all tests. All calculations and statistical analyses were performed using IBM SPSS software package for Windows (Version 20.0; IBM Corp., NY, USA).

## 3. Results

### 3.1. Changes of Thyroid Function and Lipid Profiles after THW

**Figure 1:**
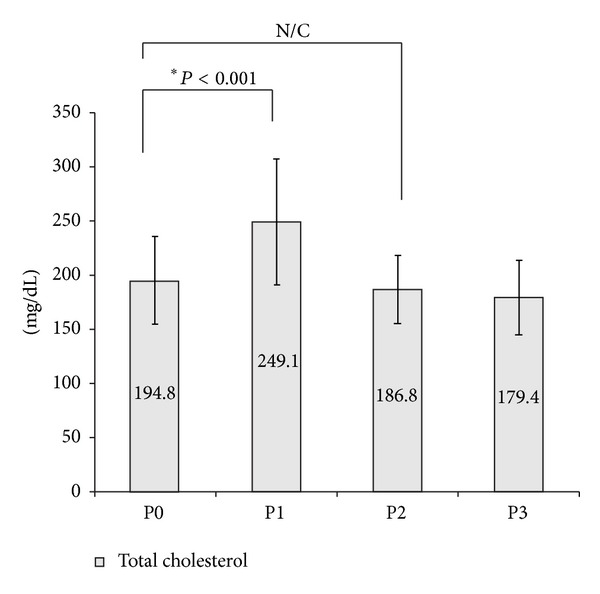
The serial change of mean total cholesterol levels.

The mean age of 61 subjects (M : F = 14 : 47) was 49 ± 11 yr, and the mean BMI was 23.9 ± 3.3 kg/m^2^. All patients showed serum TSH level greater than 30 mU/L (82.4 ± 41.3 mU/L) after a 4-week THW regimen (Table 1). We first compared TFT and lipid profiles measured across four time points (P0-3): immediate preoperative period (P0), the day of 131-I administration after THW (P1), the first follow-up visit after RRA (P2), and the second follow-up visit after RRA (P3) (Table 2). The mean total cholesterol level after THW (P1) significantly increased compared to that at preoperative baseline (P0) (249.1 ± 58.0 mg/dL versus 194.8 ± 39.8 mg/dL, *P* < 0.001), but at follow-up visit after RRA and LT-4 readministration (P2), it returned to baseline level (P0) (Figure 1). Plasma LDL-C also increased to 167.7 ± 52.0 mg/dL at P1, during which TSH elevation was also observed, but returned to within normal range thereafter. Plasma TG and HDL-C did not exceed the normal range at P1, P2, or P3. All the serum triglycerides levels measured at different three time points were below 400 mg/dL. The maximal value of triglycerides in study subjects was 340 mg/dL (P1), 239 mg/dL (P2), and 298 mg/dL (P3), respectively.

### 3.2. Correlation between BMI and Total Cholesterol Level after THW

A simple correlation analysis between post-THW total cholesterol (P1) and various clinical parameters, including BMI, age, gender, elevated TSH level after THW (L-T4-off), and preoperative baseline total cholesterol level, was performed (Table 3). As a result, preoperative baseline total cholesterol showed significant correlation with post-THW cholesterol, as expected; however, age, gender, or elevated TSH level after THW itself was not correlated with post-THW total cholesterol (P1). Interestingly, a significant correlation between preoperative BMI and post-THW total cholesterol was found (*r* = 0.263, *P* = 0.041). Post-THW total cholesterol (P1) was also associated with post-THW calculated LDL-C. The level of total cholesterol measured at follow-up visit showed correlation only at the first follow-up visit after RRA (P2).

### 3.3. Serial Changes of Correlation between BMI and Total Cholesterol before and after THW

**Figure 2:**
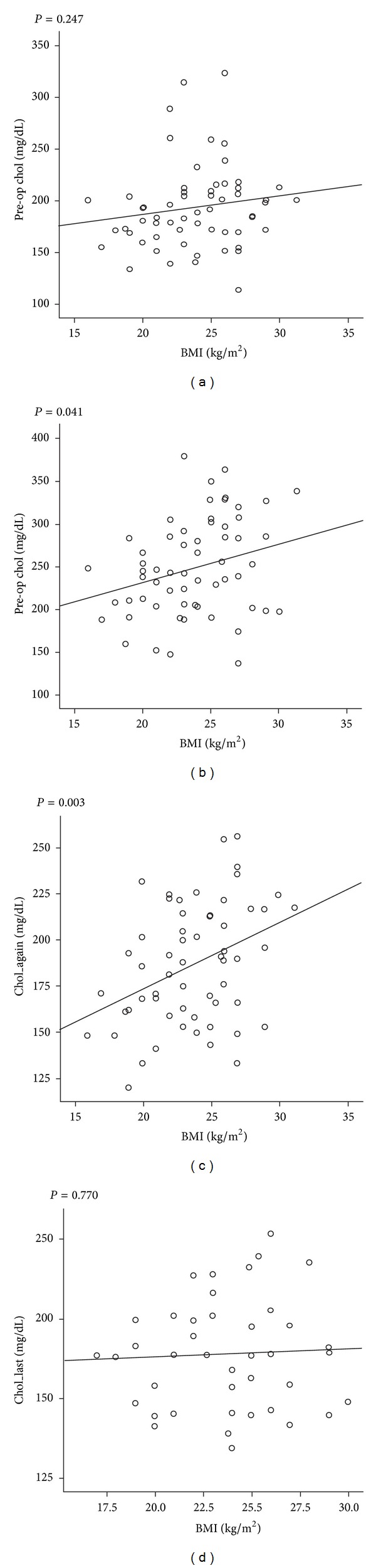
Relationship between BMI and cholesterol levels.

To further clarify the association between BMI and total cholesterol in thyroidectomized patients, the correlation between total cholesterol and BMI at each of the four time points was evaluated (Figure 2). While BMI was not correlated with total cholesterol level at baseline (P0), the correlation appeared not only at P1, but also at P2, which then disappeared at the second follow-up visit completed at 15.9 ± 3.7 months after RRA.

### 3.4. Different Changes of Total Cholesterol after THW according to BMI of 23 kg/m^2^

We stratified the patients as either normal weight (*n* = 21) or overweight group (*n* = 40) based on the criteria of BMI of 23 kg/m^2^, and their demographics, baseline biochemical parameters, and the changes of lipid profiles after THW were compared (Table 4). The overweight group was older than normal weight group (53.1 ± 9.2 vs. 39.9 ± 10.5 yr, *P* = 0.001), but there was no gender difference between the two groups. Intriguingly, the two groups did not show a significant difference in total cholesterol measured at baseline (P0) or at post-RRA follow-up visits (P2 and P3). The two groups significantly differed only in post-THW total cholesterol and LDL-C level (P1). In other words, the overweight group had the greater post-THW total cholesterol and LDL-C level compared to normal-weight group.

### 3.5. BMI as an Independent Determining Factor of Total Cholesterol Level after THW

Finally, we performed multiple logistic analyses with post-THW total cholesterol level as a dependent variable (Table 5), which found BMI to be an independent determining factor of post-THW total cholesterol level along with preoperative baseline total cholesterol level and elevated TSH level following THW (*P* = 0.012).

## 4. Discussion

Due to the rising incidence of thyroid cancers, much interest has been raised regarding the possible metabolic effects or long-term health outcomes resulting from thyroid hormonal derangement following thyroidectomy. Patients who undergo total thyroidectomy are potentially exposed to long duration of subclinical or mild hyperthyroidism due to a long-term TSH suppressive therapy; however, THW regimens required for diagnostic or therapeutic radioiodine whole body scan may induce acute thyroid hormone deficiency. Previous studies have investigated the metabolic effects caused by transient severe hypothyroidism due to THW on peripheral target organs [10, 11]. Transient alterations of lipid metabolism are one of the hallmark clinical features of such THW-induced effect, and several studies have also focused on how changes in lipid profiles under such circumstances affect the vascular function [13]. However, in clinical situations, the spectrum of THW-induced changes in lipid profile is wide: in some patients, the change is very mild, while in others, severe hypercholesterolemia is observed. This variability is also observed in patients in overt primary thyroid dysfunction [15]. Nonetheless, there has been no study directly analyzing the various associations between THW and transient dyslipidemia or elucidating the clinical factors influencing the association.

Although the effects of thyroid hormone on cholesterol metabolism are not yet fully known, it is accepted that changes in thyroid function exert a wide range of influence on the overall cholesterol metabolism, including its synthesis, metabolism, and mobilization [6]. Overt hypothyroidism is associated with tissue expression and altered activities of various regulators participating in lipid metabolism, including hepatic hydroxymethylglutaryl coenzyme A (HMG CoA) reductase, LDL-C receptors, sterol regulatory element-binding protein-2 (SREBP-2), cholesteryl ester transfer protein (CETP), hepatic lipase, lipoprotein lipase, and ATP binding cassette transporter A1 (ABCA1) transporter [16–20]. Consequently, hypothyroid patients generally show elevated total cholesterol and LDL-C level, as well as normal to elevated HDL-C, TG, and very low density lipoprotein (VLDL) [21].

There has been abundant clinical evidence on the relationship between hypothyroid status, abnormal lipid profile, and the related health outcomes such as atherosclerosis and metabolic syndrome [22–27]. On the contrary, relatively few studies have investigated the metabolic effects of acute thyroid hormone deficiency in DTC patients who have to undergo THW for postoperative 131-I therapy or for measurement of stimulated serum Tg and radioiodine whole body scan to evaluate residual/recurrent diseases [10, 11, 13]. In a prospective study on 15 female DTC patients, Chang et al. have reported that short-term hypothyroidism due to THW and the consequent worsening of metabolic parameters does not affect the measurements of surrogate marker for endothelial function, such as brachial artery diameter [13]. Nonetheless, the recent surge in number of patients with DTC, combined with mostly low mortality risk of the cases of DTC, suggests the importance of maintaining an optimal thyroid functional status in athyreotic patients through the follow-up periods with a suppressive or replacement dose of thyroid hormone, both in terms of oncologic safety and favorable metabolic health outcome. Therefore, the clinical features and metabolic effects of transient severe hypothyroid status following THW in DTC patients require further clarification.

The occurrence of hypercholesterolemia in hypothyroid patients cannot be explained with a single mechanism. A combination of results of several clinical studies has found that TSH and thyroid hormone level are both independently associated with total cholesterol level; furthermore, various clinical factors, including gender, age, fasting plasma glucose, BMI, and smoking status, are also thought to exert independent effects on total cholesterol levels [1]. In this study, we have reported that BMI is significantly associated with total cholesterol level measured after 4 weeks of L-T4 withdrawal in transiently hypothyroid, athyreotic patients independently of baseline total cholesterol level, elevated TSH level after THW, age, and gender. To our knowledge, this is the first study to have investigated the association between BMI and short-term hypothyroidism-induced hypercholesterolemia occurring during the course of postoperative surveillance in DTC.

In this study, the mean post-THW total cholesterol and LDL-C level exceeded the normal range only in overweight group of patients, defined as BMI of 23 kg/m^2^ or higher. The patients with normal body weight showed normal levels of total cholesterol and LDL-C. The baseline lipid profiles were not different between the two groups. Furthermore, while total cholesterol level returned to preoperative baseline level at the follow-up visits after RRA and resumption of L-T4 replacement, the correlation between BMI and total cholesterol level was maintained until the first follow-up visit. Since only two patients in our patients group satisfied the criteria of obesity suggested by the World Health Organization (BMI greater than 30 kg/m^2^), we have instead employed a proposed BMI cutoff for overweight (23 kg/m^2^) which takes into account the health risk for Asian population [28, 29]. In a study of 18 DTC patients who underwent THW, Huang et al. reported insulin resistance and BMI to be the factors predictive of severity of hypothyroid symptoms after THW [12]. Our study may be in line with this previous report, and we suspect that overweight athyreotic patients can be more vulnerable not only to hypothyroid symptoms but also to hypothyroidism-induced abnormality of lipid profile after THW.

Other past clinical studies have reported that insulin sensitivity may act as a modulator in the relationship between TSH and serum lipid level in healthy euthyroid subjects and female diabetic patients and insulin-resistant subjects are more susceptible to the higher risk of hypercholesterolemia as TSH level increases [30, 31]. The results of this study imply that increased hepatic cholesterol synthesis may be observed in DTC patients with high BMI due to relatively elevated insulin resistance. Hypothyroidism simultaneously suppresses hepatic cholesterol synthesis and hepatic LDL-C receptor expression: the reduced clearance of LDL-C overpowers the effects of low hepatic cholesterol synthesis in hypothyroid status, resulting in hypercholesterolemia [6]. As such, we suggest that the possible increase of baseline hepatic cholesterol synthesis following increased BMI may be the underlying reason behind the association between elevated BMI and post-THW hypercholesterolemia. However, the probable explanation for the association between BMI and post-THW hypercholesterolemia is just one of many assumptions since there was no definite evidence for the difference of insulin resistance among the subjects in this study. We could not obtain any surrogate parameters regarding metabolic status except BMI and baseline routine chemistry results. We aimed to elucidate the difference in fasting serum glucose level or uric acid level according to BMI, but it was not significant as the number of subjects was relatively small (Table 4). A larger scaled study is also required to explain the exact mechanism of the association between BMI and post-THW hypercholesterolemia.

This study has several limitations. First of all, this was a retrospective study analyzing relatively few subjects, and, therefore, a multifaceted study evaluating the effect of hypothyroidism on various aspects of lipid metabolism, including cholesterol synthesis, clearance, and efflux, must be performed in the future to further clarify the exact molecular mechanism of the effect of BMI on dyslipidemia caused by thyroid hormone deficiency in athyreotic patients. We also could not include thorough lipid profile data at time point P0 except total cholesterol level, because only routine chemistry exam was performed for preoperative blood test (P0) according to the surgical preparation protocol of our institute. The data about thyroid function tests and lipid profile at a time point between surgery and the start of thyroid hormone withdrawal also could not be analyzed in this retrospective study because they were not routinely performed in our institute. There might be some difference between baseline preoperative lipid profile and the lipid profile immediately before the start of thyroid hormone withdrawal which must be accompanied with suppressed thyrotropin level by supraphysiological levothyroxine replacement. This must be further evaluated in a prospective and more controlled study. Furthermore, the most lipid abnormalities accompanied by short-term acute hypothyroidism are reversible with L-T4 replacement, so the long-term clinical implication of this study should also be evaluated through additional prospective studies. Nonetheless, this study found a statistically significant association between BMI and post-THW hypercholesterolemia despite a small sample size of 61 patients; since THW is being more widely employed (both diagnostically and therapeutically) as part of postsurgical management of thyroid cancer, attention needs to be paid to post-THW lipid profile abnormality and its long-term health outcome, at least in patients with underlying metabolic derangements, such as overweight and insulin resistance.

In conclusions, BMI is an independent determining factor for serum cholesterol level after THW in thyroidectomized patients. Overweight patients who have undergone total thyroidectomy may be more susceptible to the adverse metabolic effects of thyroid hormone deficiency. These patients must be considered to be in higher risk of dyslipidemia when exposed to hypothyroid and athyreotic status. Maintaining BMI and thyroid hormonal status in appropriate range needs to be stressed more in this group during the postoperative follow-up periods of thyroid cancers.

## Figures and Tables

**Table 1:** Clinical and metabolic characteristics of the 61 patients.

	Mean ± SD
Sex (M/F)	14/47
Age (yr)	49 ± 11
Body mass index (kg/m^2^)	23.9 ± 3.3
Preoperative total cholesterol (mg/dL)	194.8 ± 39.8
Total cholesterol elevation (mg/dL)	54.2 ± 42.6
TSH (LT4-off) (mU/L)	82.4 ± 41.3
T3 (LT4-off) (ng/mL)	0.5 ± 0.3
Free T4 (LT4-off) (ng/dL)	0.5 ± 0.2

TSH: thyroid stimulating hormone.

**Table 2:** Serial changes of thyroid function test results and lipid profiles after thyroid hormone withdrawal.

	P0 (*n* = 61)	P1 (*n* = 61)	P2 (*n* = 61)	P3 (*n* = 41)
TSH (mU/L)	1.6 ± 1.0	82.4 ± 41.3	0.3 ± 1.1	0.2 ± 0.4
T3 (ng/mL)	1.1 ± 0.1	0.5 ± 0.3	1.1 ± 0.2	1.1 ± 0.1
Free T4 (ng/dL)	1.1 ± 0.2	0.5 ± 0.2	1.7 ± 0.4	1.5 ± 0.3
Total cholesterol (mg/dL)	194.8 ± 39.8	249.1 ± 58.0	186.8 ± 31.4	179.4 ± 34.2
Triglyceride (mg/dL)		129.8 ± 54.6	118.4 ± 56.1	115.7 ± 63.4
HDL-C (mg/dL)		56.3 ± 13.0	49.6 ± 9.6	51.1 ± 10.1
LDL-C (mg/dL)		167.7 ± 52.0	113.9 ± 28.6	103.0 ± 30.8
Follow-up duration (months)		N/C	5.9 ± 1.0	15.9 ± 3.7

TSH: thyroid stimulating hormone; HDL-C: high-density lipoprotein cholesterol; LDL-C: low-density lipoprotein cholesterol.

**Table 3:** Correlation between various clinical parameters and serum cholesterol level after thyroid hormone withdrawal.

	Cholesterol after THW	
	*r*	*P* value
BMI (kg/m^2^)	0.263	0.041
Age (yr)	0.204	0.115
Gender	0.035	0.791
TSH (LT4-off) (mU/L)	0.168	0.196
Preoperative cholesterol (mg/dL)	0.685	0.001
F/U cholesterol (1) (mg/dL)	0.274	0.036
F/U cholesterol (2) (mg/dL)	0.124	0.439
LDL-C (LT4-off) (mg/dL)	0.966	0.001

TSH: thyroid stimulating hormone; F/U: follow up; LDL-C: low-density lipoprotein cholesterol.

**Table 4:** Different serial change of lipid profile according to BMI of 23 kg/m^2^.

	BMI < 23 (*n* = 21)	BMI ≥ 23 (*n* = 40)	*P* value

Mean age (yr)	39.9 ± 10.5	53.1 ± 9.2	0.001
Sex			0.244
Female (%)	18 (38.3)	29 (61.7)	
Male (%)	3 (21.4)	11 (78.6)	
Preoperative serum glucose (mg/dL)	88.2 ± 11.8	90.2 ± 13.9	0.571
Preoperative cholesterol (mg/dL)	186.0 ± 35.0	199.4 ± 41.8	0.224
Preoperative uric acid (mg/dL)	4.4 ± 1.2	5.1 ± 1.6	0.12
ΔCholesterol (mg/dL)	40.2 ± 37.0	61.5 ± 43.9	0.068
TSH (LT4-off) (mU/L)	88.4 ± 39.7	79.3 ± 42.3	0.427
Total cholesterol (LT4-off) (mg/dL)	226.1 ± 43.8	260.8 ± 61.2	0.015
LDL-C (LT4-off) (mg/dL)	146.1 ± 36.3	178.5 ± 55.6	0.025
HDL-C (LT4-off) (mg/dL)	56.6 ± 13.5	56.1 ± 13.0	0.899
Triglyceride (LT4-off) (mg/dL)	112.6 ± 37.3	138.5 ± 60.1	0.092
F/U cholesterol (1) (mg/dL)	176.2 ± 26.8	192.6 ± 32.5	0.059
F/U cholesterol (2) (mg/dL)	179.9 ± 25.4	179.1 ± 38.4	0.951

TSH: thyroid stimulating hormone; HDL-C: high-density lipoprotein cholesterol; LDL-C: low-density lipoprotein cholesterol; F/U: follow up.

**Table 5:** Multiple logistic analyses with total cholesterol level following thyroid hormone withdrawal as a dependent variable.

Clinical parameters	Regression coefficient	*P* value
Age (yr)	−0.027	0.779
Sex	−0.02	0.82
BMI (kg/m^2^)	0.251	0.012
Preoperative cholesterol (mg/dL)	0.639	0.001
TSH (mU/L)	0.428	0.001

BMI: body mass index; TSH: thyroid stimulating hormone.

## Abbreviations

ABCA1: ATP binding cassette transporter A1
ATA: American Thyroid Association
BMI: Body mass index
CETP: Cholesteryl ester transfer protein
DTC: Differentiated thyroid cancers
HDL-C: High-density lipoprotein cholesterol
HMG CoA: Hydroxymethylglutaryl coenzyme A
LDL-C: Low-density lipoprotein cholesterol
RRA: Radioiodine remnant ablation
SREBP-2: Sterol regulatory element-binding protein-2
TFT: Thyroid function test
Tg: Thyroglobulin
TG: Triglycerides
THW: Thyroid hormone withdrawal
VLDL: Very low density lipoprotein.

## Diclosure

The authors alone are responsible for the content and writing of this paper.
